# Transcriptome Profiling of Layer 5 Intratelencephalic Projection Neurons From the Mature Mouse Motor Cortex

**DOI:** 10.3389/fnmol.2018.00410

**Published:** 2018-11-12

**Authors:** Alison J. Clare, Robert C. Day, Ruth M. Empson, Stephanie M. Hughes

**Affiliations:** ^1^Department of Biochemistry, School of Biomedical Sciences, University of Otago, Dunedin, New Zealand; ^2^Brain Health Research Centre, School of Biomedical Sciences, University of Otago, Dunedin, New Zealand; ^3^Genetics Otago, School of Biomedical Sciences, University of Otago, Dunedin, New Zealand; ^4^Department of Physiology, School of Biomedical Sciences, University of Otago, Dunedin, New Zealand

**Keywords:** *Fezf2*, low-input RNA-seq, M1, FACS, IT-PNs

## Abstract

The mature cortex contains hugely diverse populations of pyramidal projection neurons (PNs), critical to normal forebrain circuits. In order to understand the healthy cortex, it is essential to characterize this neuronal complexity. We recently demonstrated different identities for *Fezf2*-positive (*Fezf2+ve*) and *Fezf2*-negative (*Fezf2−ve*) intratelencephalic-PNs (IT-PNs) from layer 5 of the motor cortex (M1). Comparatively, each IT-PN type has a distinct electrophysiological phenotype and the *Fezf2+ve* IT-PNs display a unique apical dendritic tuft. Here, we aimed to expand our understanding of the molecular underpinnings defining these unique IT-PN types. Using a validated *Fezf2*-GFP reporter mouse, retrograde labeling techniques and fluorescence activated cell sorting (FACS), combined with a novel approach for low-input RNA-sequencing, we isolated mature *Fezf2+ve* and *Fezf2−ve* IT-PNs for transcriptome profiling. Through the comparison of *Fezf2+ve* and *Fezf2−ve* IT-PN gene expression profiles, we identified significant enrichment of 81 genes in the *Fezf2+ve* IT-PNs and 119 genes in the *Fezf2−ve* IT-PNs. Term enrichment analysis of these enriched genes demonstrated significant overrepresentation of the calcium-binding EF-hand domain in *Fezf2+ve* IT-PNs, suggesting a greater importance for calcium handling in these neurons. Of the *Fezf2−ve* IT-PN enriched genes an unexpected and unique enrichment of genes, previously associated with microglia were identified. Our dataset identifies the molecular profiles of two unique IT-PN types in the mature M1, providing important targets to investigate for their maintenance in the healthy mature brain.

## Introduction

Thecerebral cortex of the brain is a layered structure essential for higher cognitive function. Here, there are large numbers of projection neurons (PNs) important for encoding the messages that drive our thoughts and actions (Spruston, 2008). Commonly PNs are grouped into subtypes according to their projection type (e.g., to the contralateral cortex or corticospinal tract; Molyneaux et al., 2007). However, analyses have shown much greater diversity in PN types, both across and within layers of the cortex (Hattox and Nelson, 2007; Oswald et al., 2013; Rouaux and Arlotta, 2013; Tantirigama et al., 2016). For example, recent analyses of intra-telencephalic projection neurons (IT-PNs) within layer 5 of the mature mouse primary motor cortex (M1) identified two unique subtypes that were defined by the expression or absence of transcription factor *Fezf2* (Tantirigama et al., 2014). Characterization of the unique genetic profiles that underpin such neuronal diversity is vital to our understanding of their maintenance in a healthy adult brain.

Several recent studies have applied the concept of separating cell-types, such as cortical PNs (Arlotta et al., 2005; Molyneaux et al., 2009, 2015) and striatal PNs (Lobo et al., 2006), from the mouse brain to identify cell-type specific gene expression. Important contributions have been made from this work, including identification of the transcriptional regulatory networks driving cortical development (Arlotta et al., 2005) and transcription factors essential to the differentiation of striatonigral neurons (Lobo et al., 2006). However, broad grouping of neurons, for example based on their projections, can mask gene expression unique to distinct subtypes or even individual neurons, a fact that is quickly being realized with the recent advancement of low-input and single-cell RNA-sequencing technologies (Darmanis et al., 2015; Usoskin et al., 2015). To expand our grasp on neuronal subtype gene expression, greater separation of cell types will be essential.

The recent analysis of layer 5 PNs in the mature M1, revealed clear separation of IT-PN types according to the expression of the developmentally important transcription factor *Fezf2* (Tantirigama et al., 2014). Characterization of the *Fezf2+ve* and *Fezf2−ve* IT-PNs revealed distinct morphological and functional phenotypes, hinting at a unique role for these neurons in the cortical circuitry (Tantirigama et al., 2014). For, example, *Fezf2+ve* IT-PNs have a unique apical tuft extending through upper layers of the cortex, which is absent in *Fezf2−ve* IT-PNs. The micro-circuitry inputs to M1 demonstrate sublayer specificity, sensory inputs (sensory thalamus and somatosensory cortex, S1) targeting upper layers (2/3 and 5A; Mao et al., 2011; Hooks et al., 2013), whilst inputs from motor thalamus can also directly target pyramidal tract PNs (PT-PNs; Hooks et al., 2013), found in deeper layers of the cortex. Therefore, the morphological differences identified in IT-PNs of layer 5 suggest differing contributions to the micro-circuitry, with *Fezf2+ve* IT-PNs more likely to receive inputs from upper layers of the cortex. Based on the divergence in *Fez2+* and *Fezf2–* phenotypic features we sought to investigate the differential gene expression that defines these two IT-PN types.

In the work presented here, we labeled IT-PNs in a *Fezf2-Gfp* reporter mouse model with a fluorescent retrograde tracer to allow FACS purification of *Fezf2+ve* and *Fezf2−ve* IT-PNs from layer 5 of M1. In this work we applied a combined PCR pre-amplification and *in vitro* transcription (IVT) method to amplify RNA to sequence the transcriptomes from very low RNA-input (Day et al., 2018). Our cDNA library preparation method utilizes unique barcodes in the initial cDNA synthesis stages, a common tool in low-input methods (Hashimshony et al., 2012; Islam et al., 2014). The advantage of barcoding is the ability to pool samples, creating a greater yield in starting material, important for efficient IVT amplification (Hashimshony et al., 2012). Before final library preparation amplified RNA (aRNA) is fragmented to produce a 3’ bias “tag-like” library, which simplifies normalization strategies during later analyses, as gene length does not need to be considered (Hashimshony et al., 2012). Utilizing this method, we found clear separation of *Fezf2+ve* and *Fezf2−ve* IT-PN types according to their molecular profiles, rather than the host animal from which they came. Furthermore, we identified the unique expression of several molecular factors that could contribute to their functional and morphological differences.

## Materials and Methods

### Mice

All experiments were performed using male Swiss-Webster mice of either wild-type (non-transgenic) or hemizygous transgenic (Zfp312-EGFP)CO61GsatMmnc mouse line (Gong et al., 2003) bred on a Swiss Webster background strain. The hemizygous transgenic mice express a GFP reporter gene under the control of *Fezf2* regulatory elements (referred to from here on as *Fezf2-Gfp* mice). Male mice were selected for this study as it continues the comparison of IT-PN types, previously identified in work by Tantirigama et al. (2014), where only male mice were used. The University of Otago Animal Ethics Committee approved all animal husbandry, surgical procedures and use of animal tissue (AUP 110/13).

### Stereotaxic Surgeries for the Injection of CTB647

For the injection of retrograde tracer, CTB647 (0.35% w/v in saline; Life Technologies, New Zealand) male *Fezf2-Gfp* mice (P23–25) were anesthetized by a sub-cutaneous injection of 0.05 mg/kg atropine (Baxter Healthcare Ltd.), 0.5 mg/kg domitor (Novartis NZ Ltd., New Zealand) and 70 mg/kg ketamine (Parnell Laboratories NZ Ltd.) before securing them onto stereotaxic equipment using 45°C non-rupture ear bars and inserting the nose clamp gently into the mouth (310037R, Kopf Instruments). The CTB647 was prepared at 0.35% (w/v) in saline and 0.5 μL delivered through a craniotomy in the skull using a 10 μL Nanofil syringe and 33 G needle assembly (World Precision Instruments, Sarasota, FL, USA). The CTB647 retrograde tracer was delivered to two injection sites in the left hemisphere primary motor cortex; +0.85 mm anterior from Bregma, +1.65 mm lateral from midline and −0.85 mm depth from the pial layer and +0.4 mm anterior from Bregma, +1.4 mm lateral from the midline and −0.6 mm depth from the pial layer.

### Histology

Five to seven days after injection of the CTB647 tracer, animals were anesthetized with 150 mg/kg pentobarbital before intracardial perfusion with 20 mL ice cold 0.9% (w/v) NaCl followed by 20 mL ice cold 4% (w/v) paraformaldehyde (PFA) in 0.1 M phosphate buffer (pH 7.2). Tissue was post-fixed overnight in ice cold 4% PFA at 4°C, followed with cryoprotection in 30% sucrose for 2–3 days at 4°C. Brain tissue was frozen in optimal cutting temperature medium (OCT), Tissue-Tek^®^ (Thermo Fisher Scientific, New Zealand) before cutting 40 μm thick coronal sections at −20°C using a Leica CM3050 cryostat (Leica Biosystems, Richmond, IL, USA). Tissue was mounted on Superfrost™ Plus slides (Thermo Fisher Scientific, New Zealand) in anti-fade solution (0.1 g *p*-phenylenediamine made with 80% (v/v) glycerol in 0.1 M phosphate buffer, pH 8.5). Images were captured on an Olympus inverted microscope IX71 and Adobe Photoshop used to merge images.

### Immunohistochemistry and Image Analysis

Free-floating *Fezf2-Gfp*, CTB647 labeled sections were blocked for 1 h at room temperature in PBS with 0.2% (v/v) triton-X and 3% (v/v) normal goat serum. Sections were incubated with an antibody raised against ryanodine receptor 2 (RYR2; NBP1-90091, rabbit polyclonal; Novus Biologicals, Littleton, CO, USA) for 36 h at 4°C before washing three times in PBS-T for 10 min each. The primary antibody was labeled with a rabbit Alexa 594 secondary (A11037; Invitrogen, New Zealand) for 4 h at room temperature. Sections were washed three times in PBS-T for 10 min each before mounting on Superfrost™ Plus slides (Thermo Fisher Scientific, New Zealand) in Anti-fade Gold (Life Technologies, New Zealand).

Confocal laser scanning microscopy was performed using the Nikon C2si+ platform, with a 20× (NA 0.75) objective and a single plane image captured. ImageJ (Schneider et al., 2012) was used to apply a threshold to images captured of RYR2 immunostaining. Individual cell bodies boundaries were identified according to overlay with CTB and Fezf2-GFP staining. Using ImageJ, a region of interest was then selected around individual cell bodies of the RYR2 threshold image and the % area of pixels quantified. Quantification was performed on 8–10 cells of either *Fezf2+ve* or *Fezf2−ve* IT-PN types (identified according to GFP and CTB647 labeling) in 5–6 sections per animal. For each image the % area of pixels/cell was normalized to the mean % area pixels of all *Fezf2+ve* IT-PNs. Data for each animal is presented as the average in normalized % area of fluorescence across sections (± standard error of the mean, SEM; *n* = 3 mice). A paired *t*-test was used to analyze differences in average relative RYR2 expression of *Fezf2+ve* and *Fezf2−ve* IT-PNs.

### Microdissection of Layer 5 M1

Pentobarbitol (150 mg/kg) was used to anesthetize animals before intracardial perfusion with ice cold 0.9% (w/v) NaCl. A Ted Pella acrylic mold (Ted Pella Inc. Redding, CA, USA) was used to make 1 mm coronal sections of the adult M1 from Bregma +1.2–0. The brain slices were transferred to L15 complete media; phenol red free Leibovitz’s L15, 6% D-(+)-glucose (Sigma Aldrich, New Zealand) and 500 units penicillin/500 μg streptomyocin per mL (Invitrogen, New Zealand). Layer 5 was microdissected from the right hemisphere, according to GFP expression, using the Olympus SZX12 with a GFP optic cube. The dissected tissue was processed immediately into single cell suspension.

### Cell Dissociation

Dissected samples were diced finely in L15 complete media and transferred to a 2 mL eppendorf tube. After tissue settled, L15 media was removed and replaced with digestion media; 12 U/mL papain (Worthington Biochemical Corporation, Lakewood, NJ, USA) and 1 U/mL DNase I (Invitrogen, New Zealand) in L15 complete media, and rotated on MACS mixer (Miltenyi Biotec, Sunnyvale, CA, USA) for 15 min at 37°C. Digestion media was then replaced with blocking/trituration solution (2% B27, 1 U/mL DNase I; Invitrogen, New Zealand) and incubated for 10 min at 37°C with 5% CO_2_. After resuspending the samples in fresh blocking/trituration media, tissue was passed through a series of decreasing bore size, fire-polished Pasteur pipettes. The dissociated cells were pelleted by centrifugation at 1,000× *g* for 5 min and resuspended in ice cold L15 complete media. DAPI (0.1 μg/mL) was added to cell resuspension 5 min prior to cell sorting.

### Cell Sorting

A FACSAria with BDFACSDiva software (BD Biosciences, Australia) and a 70 μm nozzle was used to sort cells. Dissociated samples were analyzed to determine the parameters for sorting and gates were set according to the physical light scatter patterns (FSC and SSC; Supplementary Figure S1). The events with high granularity (SSC) and small in size (low FSC) are debris (Guez-Barber et al., 2011) and were gated out. Prior to sorting the fluorescently labeled samples, wild-type Swiss Webster tissue was analyzed to determine autofluorescence and to set the parameters (gates) for isolating cells (Supplementary Figure S1). Cells were collected either according to the gates set based on physical profiles (elimination of debris and doublets; wild-type samples), or according to their fluorescent profiles (*Fezf2-Gfp* and Alexa 647). Excitation for GFP was from a 488 nm laser with detection from the 530/30 filter; excitation of CTB647 was from a 633 nm laser with detection from 660/20 filter and excitation of DAPI from a 407 nm laser with detection from 450/40 filter. All cells were sorted from a live, DAPI negative population (Supplementary Figure S1) and were sorted directly into RNA lysis buffer from the RNAqueous micro kit (Invitrogen, New Zealand). At least 20,000 events were recorded during the sort for setting the sorting criteria and for post-sort analysis. This data was analyzed using Flowing software (Cell Imaging Core, Turku Centre for Biotechnology, Finland) and representative graphs were made using Flowjo (Treestar, Woodburn, OR, USA). For each sample ≥3,000 cells were sorted.

### RNA Extraction and QC

RNA was extracted using the RNAqueous micro RNA extraction kit (Invitrogen, New Zealand), according to the manufacturer’s instructions. RNA was eluted in two repeated steps as follows; 5–10 μL of elution solution, preheated to 75°C, was added to the cartridge and incubated for 1 min at room temperature before centrifugation for 30 s. Eluted RNA was then DNase I treated, according to manufacturer’s instructions, with the included RNAqueous micro RNA extraction kit reagents. Quality and quantity of RNA was assessed with the Agilent TM RNA 6,000 Pico assay on an Agilent 2,100 bioanalyzer. Ribosomal peaks were detected, using the Agilent™ Bioanalyzer, in RNA isolated from cell fractions as small as 6,000 cells, however yields were too low to accurately determine the RNA quality. Larger cell fractions (>20,000) were used to determine the RNA integrity number (RIN), with a range in RIN values of 6.5–8.3.

### cDNA Library Generation and RNA-Sequencing

Three microliters of RNA (equating to an input of 400–3,000 cells) was denatured at 72°C for 3 min with 33 ng barcoded oligo dT primer (Supplementary Table S1), 4.4 mM dNTPs (Roche, New Zealand), 2 ng of ERCC spike in mix 1 (Life technologies, New Zealand) and 10 U RNase inhibitor (Enzymatics, Beverly, MA, USA). Two microliters of this ERCC-oligo-RNA mix was then added to a 3 μL mix including; 1× PrimeScript™ reaction buffer 0.5 μL PrimeScript™ reverse transcriptase enzyme mix (Takara Bio Inc. Japan), 5.6 mM MgCl_2_ (Roche, New Zealand), 935 mM Betaine (Sigma Aldrich, New Zealand) 2.3 mM DTT and 0.56 μM template switch oligos (TSOs; Supplementary Table S1). First strand cDNA synthesis was then performed with the following protocol; 25°C (5 min), 42°C (90 min), 70°C (15 min), 4°C (hold). The cDNA pool was PCR pre-amplified in a 20 μL reaction using 1× HiFi Kapa PCR mix (Kapa Biosystems Inc., Wilmington, MA, USA), 0.5 μM of primers designed to the sequence within the TSO oligos and to the sequence within the oligodT (Supplementary Table S1). The PCR protocol was as follows; 98°C (3 min) followed by 20 cycles of 98°C (20 s), 70°C (15 s), 72°C (6 min) and a final extension at 72°C (5 min). Agencourt AMPure XP beads (Beckman Coulter, CA, USA) were then used at a 0.6:1 ratio to purify the double-stranded cDNA (Picelli et al., 2014), before quantification using the high-sensitivity (HS) DNA assay on the Qubit^®^ (Life Technologies, New Zealand), according to manufacturer instructions.

RNA was amplified (aRNA) from the double-stranded cDNA using the MessageAmp™ II aRNA amplification kit (Life Technologies, New Zealand). The cDNA pre-amplified samples were combined at equal ng amounts each to total 10 ng of cDNA input and added to the reaction master mix; 4 μL T7 ATP, 4 μL T7 CTP, 4 μL T7 GTP, 4 μL T7 UTP, 4 μL T7 enzyme and 1× T7 reaction buffer, before incubating at 37°C for 14 h. Sixty microliters of nuclease-free water was then added before aRNA purification using the RNeasy^®^ MinElute^®^ clean-up kit (Qiagen, New Zealand). RNA was quantified using the high sensitivity Qubit^®^ RNA assay kit (Life Technologies, New Zealand).

The final cDNA libraries were prepared as follows; all aRNA was treated with 4 U TURBO™ DNase (Ambion, New Zealand) and incubated at 37°C for 15 min before adding 39 μL of UltraPure Distilled Water (Invitrogen, New Zealand). aRNA was then fragmented with 9 μL of Fragmentation buffer (10×; NEBNext^®^ Magnesium RNA Fragmentation, New England Biolabs, New Zealand) and incubated at 94°C for 90 s. Ten microliters of RNA fragmentation stop solution was added and the reaction incubated on ice. The reaction was made up to 100 μL before proceeding to purify the fragmented aRNA using a RNeasy^®^ MinElute^®^ kit.

The fragmented aRNA was reverse transcribed in a reaction with; 2 μL aRNA (~1 μg), 4 μL nuclease-free H2O, 1× PrimeScript™ reaction buffer, 1 μL of PrimeScript™ reverse transcriptase enzyme mix and 1 μL oligo (Second_RT; Supplementary Table S1) using the following protocol; 25°C (5 min), 37°C (30 min) and 85°C (1 min). The resulting first strand cDNA was purified using AMPure XP beads before PCR amplification. The cDNA was added to a master mix of 1× HiFi Kapa PCR mix, 0.5 μM of forward and reverse primer (Supplementary Table S1) and the following PCR protocol was used; 98°C (3 min) followed by six cycles of 98°C (30 s), 65°C (1 min) and 72°C (90 s).

Libraries were sequenced on a MiSeq desktop sequencer using the MiSeq reagent kit v3 (Illumina).

### Bioinformatics

Reads were demultiplexed according to the inline barcode using sabre^1^ before trimming to remove poorer quality sequence at the ends and any remaining polyA tail contamination using the FASTX-Toolkit (Cold Spring Harbor Laboratory, Cold Spring Harbor, NY, USA). After trimming any read <50 nt was discarded. Reads were mapped to ERCC sequences (Life Technologies, New Zealand) or the mouse genome build mm10 using tophat2 (Kim et al., 2013). The resulting alignment BAM files were sorted and indexed using SAMTools (Li et al., 2009). Region coverage was assessed using both RNA-seq QC plot feature in SeqMonk (Babraham Institute, UK) and Picard’s RnaSeqMetrics available in BaseSpace (Illumina). The mean from both applications was used for plotting region-mapping statistics.

The University of California Santa Cruz (UCSC) table browser (Karolchik et al., 2004) was used to download RefFlat annotations in BED format. ERCC annotations were acquired from ERCC Controls Annotations: ERCC RNA Spike-in Control Mixes (Thermo Fisher 2016). Assignment of reads counts was performed using BEDTools multiCov (Quinlan and Hall, 2010).

DEseq2 (version 1.10.1; Love et al., 2014) was used in R studio (R version 3.2.5) for the analysis of differential gene expression. A multi-factor design was used in order to consider the pairing of samples (i.e., group; animal 1–5 and condition; GFPCTB vs. CTB only). Genes were filtered using edgeR (Robinson et al., 2010), leaving only those with ≥10 counts per million in at least 2 samples for differential expression analysis. Genes were considered differentially expressed genes with a false discovery rate (FDR) cut-off ≤0.1. All genes within this cut-off had a log fold-change ≥2.

For unsupervised hierarchical clustering of samples, genes were first ranked according to their variance across all samples, irrespective of sample type. The top 8% most variably expressed genes were then selected for clustering analysis, to remove impact of random variance. The R package pvclust, which calculates an approximately unbiased (AU) and bootstrap (BP) *p*-value for each cluster, was used to cluster the highly variable genes (Suzuki and Shimodaira, 2006). Heatmap generation was performed as described in Clare et al. (2017).

For Gene Ontology (GO) analysis of top expressed genes in our dataset and comparison to previous single cell datasets, the expression data of layer 5 neurons or microglia were obtained from linnarssonlab.org/cortex (Zeisel et al., 2015). GOrilla (Eden et al., 2009) was used to identify enriched terms, with all data sets analyzed on a background gene list obtained through combining the datasets and removal of low-counts (>10 cpm in at least five samples).

### Linear Regression Analysis of External RNA Controls Consortium (ERCC)

R studio was used for linear regression analysis and to plot graphs. Any ERCC sequences that had 0 counts for all samples were removed prior to normalization and transformation of counts. The ERCC spike-in raw counts were normalized as cpm and transformed (log2) for each sample and the average taken for linear regression analysis. The number of molecules spiked in was calculated based on the attomoles/μL for ERCC spike in mix 1 (Thermo Fisher) and the reaction dilution.

### Statistical Analysis of Mapping Rates

To analyze the differences in the proportion of mapping to exonic, intronic and intergenic regions, a two-way ANOVA was used. As this two-way ANOVA showed significant interaction a Tukey’s *post hoc* multiple comparisons test was applied to identify differences in exonic, intronic or intergenic regions between groups. The statistics presented are the result of the multiple comparisons test.

### Term Enrichment Analysis

Term enrichment analysis was performed using the online Database for Annotation, Visualisation and Integrated Discovery (DAVID v6.7) tool: functional annotation (Huang et al., 2007). All genes expressed across the samples were uploaded as the background gene list. The list of differentially expressed genes were separated into two lists, those increased within GFP-positive IT-PNs and those increased in expression within the GFP-negative IT-PNs. Each list was uploaded as a target list gene separately for analysis of enrichment. The categories databases included for term enrichment analysis; GO (biological pathway, cellular component and molecular function), KEGG (Kyoto Encyclopedia of Genes and Genomes) Pathway, InterPro and Protein information resource (PIR) superfamily. The classification stringency was set to high and the EASE score set to 0.5. Enrichment scores were assigned to annotation clusters. These scores are the accumulative geometric mean of the EASE score (a modified Fisher’s exact test in which multiple correction issues are considered; Huang et al., 2007) and displayed as –log10 of the *p*-value (e.g., a score of ≥1.3 is equal to a *p*-value ≤0.05).

### Quantitative PCR

The PCR pre-amplified cDNA from the IT-PN samples (*n* = 3 or 4 of each *Fezf2+ve* and *Fezf2−ve* samples) was analyzed for gene expression using the Roche LightCycler^®^ 480 and SYBR green method (Roche, New Zealand). Briefly each reaction included; 500 nM of each forward and reverse primer (Supplementary Table S2), 1× LightCycler 480 SYBR Green I master mix (Roche, New Zealand) 3 μL of cDNA (diluted 1:9 in RNase/DNase free H_2_O) made to 10 μL with RNase/DNase free H_2_O. Due to low sample availability, the qPCR was run in duplicate in a LightCycler^®^ 480 Multiwell −96 or −384 plate (Roche, New Zealand). A H_2_O negative control reaction was included on each plate for each primer set. The amplification protocol was as follows; 95°C for 5 min followed by 50 cycles of 95°C (5 s), 60°C (5 s), 72°C (10 s). For relative quantification, expression was normalized to two reference genes (*Wdr33* and *Rplp0*) based on the observation of stable expression in the RNA-seq. In order to analyze the expression of *Wdr33* and *Rplp0, Gapdh* was included as the second reference gene. Relative quantification was calculated using the Pfaffl efficiency method (Pfaffl, 2001).

### RT-PCR of Endogenous *Fezf2* Expression

To analyze endogenous *Fezf2* expression in sorted IT-PNs from the *Fezf2-Gfp* mouse, the primers and protocols described in Tantirigama et al. (2016) were used.

## Results

### Retrograde Labeling and FACS-Purification to Isolate *Fezf2*-Positive and *Fezf2*-Negative IT-PNs

**Figure 1:**
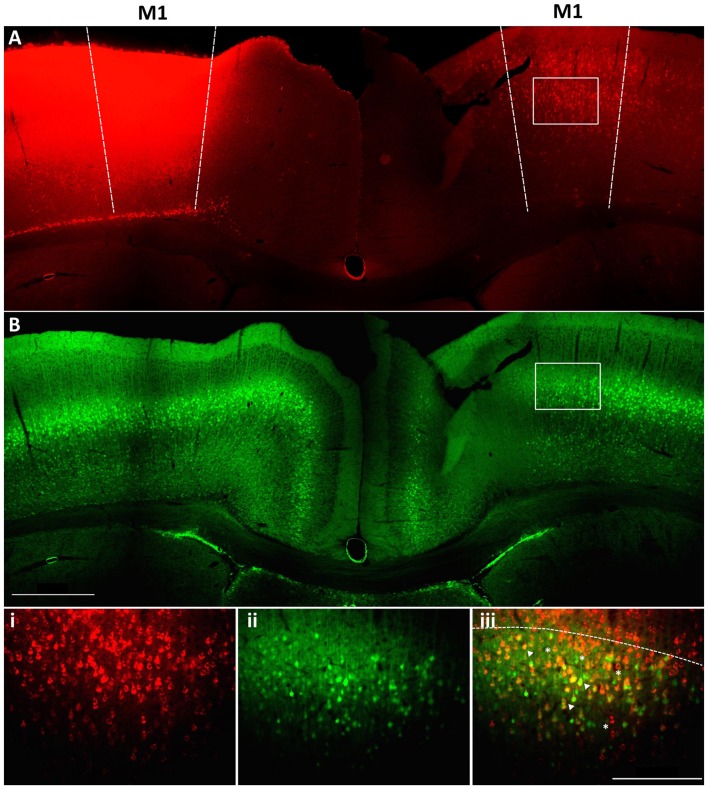
Retrograde labeling to target *Fezf2+ve* and *Fezf2−ve* IT-PNs in M1. A cholera toxin subunit b retrograde tracer conjugated to Alexa 647 (CTB647) was injected into the left hemisphere M1 of a *Fezf2-Gfp* mouse. **(A)** Image shows neurons labeled with retrograde tracer (red). **(B)** *Fezf2*-GFP expression in the same section (green). White box indicates area taken at higher magnification **(i)** CTB647 labeling, **(ii)** GFP expression, **(iii)** overlay of GFP and CTB647. Neurons in the contralateral hemisphere were labeled with CTB647, including *Fezf2*-GFP positive (arrowhead) and *Fezf2*-GFP negative (asterisk) neurons in layer 5. Dashed line in bottom panel indicates layer 2/3 and layer 5 boundary. Image was taken at Bregma 0.4. Scale bar is 500 μm or 200 μm (bottom panel).

Within M1 layer 5 of the mature brain, *Fezf2* defines two very distinct subtypes of IT-PN (Tantirigama et al., 2014). Here, we wanted to investigate what transcriptome differences could underpin two such distinct IT-PN types that reside together in the brain. In order to profile these two subtypes individually we needed to isolate the cells from M1 tissue. Previously, we have injected a retrograde tracer CTB conjugated to fluorophore Alexa 647 (CTB647) in the *Fezf2-Gfp* M1 to label *Fezf2+ve* and *Fezf2−ve* IT-PNs in layer 5 of the contralateral hemisphere (Tantirigama et al., 2014, 2016), assigning a fluorescent profile to these cells. We confirmed that injection of the tracer at two sites in M1 could label GFP(*Fezf2*)+ and GFP(*Fezf2*)- IT-PNs in the opposite hemisphere of the *Fezf2-Gfp* M1 (Figure 1). Live fluorescent labeling of these IT-PN types therefore allowed for FACS-purification of cells from a dissected layer 5 (Figure 1iii).

**Figure 2:**
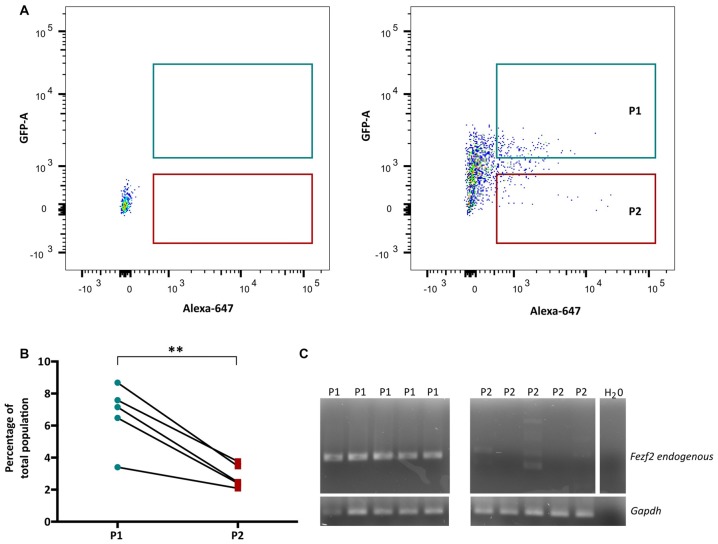
*Fezf2+ve* and *Fezf2−ve* intratelencephalic projection neurons (IT-PNs) are separated according to their fluorescence profiles. **(A)** Representative plot of Alexa 647 (CTB647; x-axis) and GFP detection (y-axis) during FACS. The fluorescence profile of a wildtype animal was used to set the gates for GFPCTB+ (GFP and CTB646; P1) and CTB+ (CTB647; P2) positive cells during sorting. **(B)** Flow cytometry analysis of cells gated as GFPCTB+ or CTB+. The percentage of GFPCTB+ neurons was significantly greater that CTB+ neurons (**p = 0.005, paired *t*-test; *n* = 5). **(C)** RT-PCR analysis of *Fezf2* expression in sorted samples. A specific (324 bp) *Fezf2* product was detected in GFPCTB+ (*Fezf2+ve*) IT-PNs (P1), but not CTB+ (*Fezf2−ve*) IT-PNs (P2). Housekeeping gene *Gapdh* was used as a positive control and detected in all samples (190 bp product).

In order to isolate the two IT-PN populations, the fluorescent profiles of cells were analyzed, plotting Alexa 647 (CTB647) against GFP and conservative gates were set according to the fluorescent profile of a wildtype (non-fluorescent) sample (Figure 2A). The percentage of GFPCTB+ cells averaged at 6.7% (±0.89, *n* = 5), which was significantly greater than the percentage of CTB+ only cells (2.84% ± 0.33 *n* = 5; *p* = 0.005, paired *t*-test; Figure 2B). This was reflected in the total number of cells collected for each IT-PN type, with an average of 11,280 (±3,810, *n* = 5) GFPCTB+ cells and 4,544 (±1,202, *n* = 5) CTB+ cells collected.

We have previously shown that endogenous *Fezf2* is detected from a total GFP+ neuronal population isolated from the *Fezf2-Gfp* mouse, validating the reliable reporting of *Fezf2*-expression in this reporter mouse model (Tantirigama et al., 2016). However, *Fezf2* is expressed in diverse populations of neuronal subtypes in mouse M1 (Tantirigama et al., 2016) and this analysis did not separate the neuronal subtypes for individual interrogation of endogenous *Fezf2* expression. Therefore, before RNA-sequencing analysis of the two IT-PN types, RT-PCR was performed to validate the correct separation of these neuronal cell types and determine that *Fezf2* is endogenously expressed in the *Fezf2-Gfp* IT-PNs. Accordingly, the endogenous *Fezf2* amplicon was detected in the GFPCTB+ cell fractions (*n* = 5), whilst there was no amplification of the *Fezf2* product in the CTB+ only samples (*n* = 5; Figure 2C; Supplementary Figure S3).

### Analysis of cDNA Library Quality and RNA-Sequencing

The small numbers of IT-PNs isolated during FACS-purification led to low yields of RNA. In order to perform RNA-seq analysis on these samples, it was therefore necessary to amplify RNA for the generation of cDNA libraries. We used a low RNA input approach to library generation on our isolated neuron samples, which includes both a PCR pre-amplification (Picelli et al., 2014) and IVT amplification (Hashimshony et al., 2012) of the RNA sample. Previous development of this tool has demonstrated robust analysis of gene expression to a single-cell level, successfully delineating different cell types in a mix of PC3 and HeLa cell lines (Day et al., 2018). Our analysis here of a control synthetic RNA spike in (ERCC) showed a significant positive relationship between ERCC coverage from RNA-seq data and the known molecules spiked in (*R*^2^ = 0.914, *p* < 2.2e-16; Supplementary Figure S2), demonstrating that, despite the heavy amplification, there is still an accurate depiction of a known input.

In order to establish the suitability of the low-input RNA-seq method on our samples, we first analyzed a number of metrics that indicate quality of library generation (Adiconis et al., 2013). After trimming and filtering, each sample had between 140,000 and 300,000 reads from sequencing. When mapping these reads back to the mouse genome the average mapping rate was 80.3% (± 6.8, *n* = 10; Table 1). Analysis of the libraries revealed low duplication rates (8.8% ± 0.9, *n* = 10), suggesting a good level of library complexity.

**Table 1:** Mapping rate of RNA-sequencing reads from *Fezf2+ve* and *Fezf2−ve* IT-PN samples (number indicates sample pair).

	Number of processed reads	Number of reads mapped to ERCC	Number of reads mapped to mm10	% of reads mapped to mm10 (post-ERCC read filter)
*Fezf2+ve 1*	303,908	103,498	133,839	66.8
*Fezf2+ve 2*	138,515	10,702	104,034	81.4
*Fezf2+ve 3*	139,117	8,130	110,522	84.4
*Fezf2+ve 4*	200,829	21,938	152,740	85.4
*Fezf2+ve 5*	257,551	18,928	200,475	84.0
*Fezf2−ve 1*	290,156	55,339	167,670	71.4
*Fezf2−ve* 2	153,010	33,131	90,147	75.2
*Fezf2−ve* 3	207,857	11,768	161,652	82.4
*Fezf2−ve 4*	266,148	22,999	207,411	85.3
*Fezf2−ve 5*	231,996	6,251	195,223	86.5

**Figure 3:**
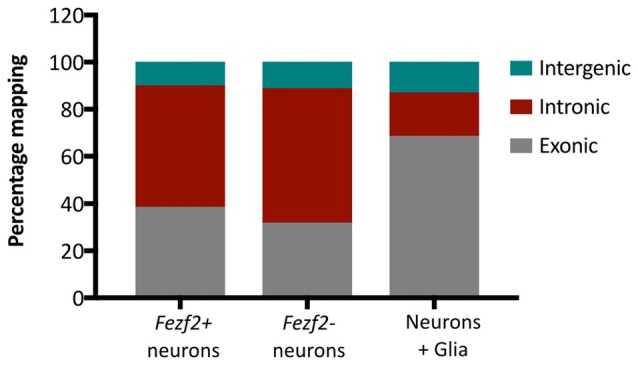
Neuronal mRNA displays increased mapping to intronic regions. Graph shows the mean percentage mapping of reads to exonic, intronic and intergenic regions for mixed neural cells, *Fezf2+ve* IT-PNs and *Fezf2−ve* IT-PNs. Compared to the mixed neural cells there was a significant increase in the mapping of reads to intronic regions from the *Fezf2+ve* IT-PN samples (*p* = 0.0004; two-way ANOVA) and *Fezf2−ve* IT-PNs (*p* < 0.0001, two-way ANOVA) and a significant decrease in mapping to exonic regions (two-way ANOVA; *Fezf2+ve* IT-PNs, *p* = 0.0012; *Fezf2−ve* IT-PNs, *p* = 0.0001). No significant differences were observed for the mapping rates to intergenic regions across all samples and no differences were observed between IT-PN samples.

Additionally, we analyzed the proportion of reads mapping to different genomic regions to determine transcript composition. There was no excessive mapping to intergenic regions (10.6% ± 1.2, *n* = 10), with rates similar to those observed in the literature for low RNA input cDNA libraries (Adiconis et al., 2013), indicating minimal contamination from genomic DNA in the library preparations. However, we observed high rates of mapping to intronic sequences in both the *Fezf2+ve* (52% ± 7, *n* = 5) and *Fezf2−ve* (57% ± 5, *n* = 5) IT-PNs, with the largest mapping rate to introns in an individual sample reaching 67%. It has previously been shown that pyramidal cortical neurons retain a similarly high proportion of non-coding regions (Dueck et al., 2015). Therefore, to confirm that the observation of high intronic sequence retention was unique to isolated neurons, we sorted a collection of unspecified cell bodies (mixed neuronal and glia) from wildtype M1 tissue for RNA-seq analysis. Mapping rates to intergenic regions were no different in the mixed samples compared to the IT-PN data. However, the mapping rates to exonic regions were significantly increased when compared to both *Fezf2+ve* (p ≤ 0.01, *n* = 5, 5, 3) and *Fezf2−ve* (*p* < 0.001, *n* = 5, 5, 3) neurons and mapping to intronic regions was significantly decreased (*Fezf2+ve* vs. mixed, *p* < 0.001; *Fezf2−ve* vs. mixed, *p* < 0.0001, *n* = 5, 5, 3; Figure 3; Supplementary Table S3). Thus, it could be concluded that the RNA biology of a neuron-enriched population appears to retain a higher proportion of intronic regions.

### IT-PN Types Are Molecularly Distinct According to *Fezf2* Expression

**Figure 4:**
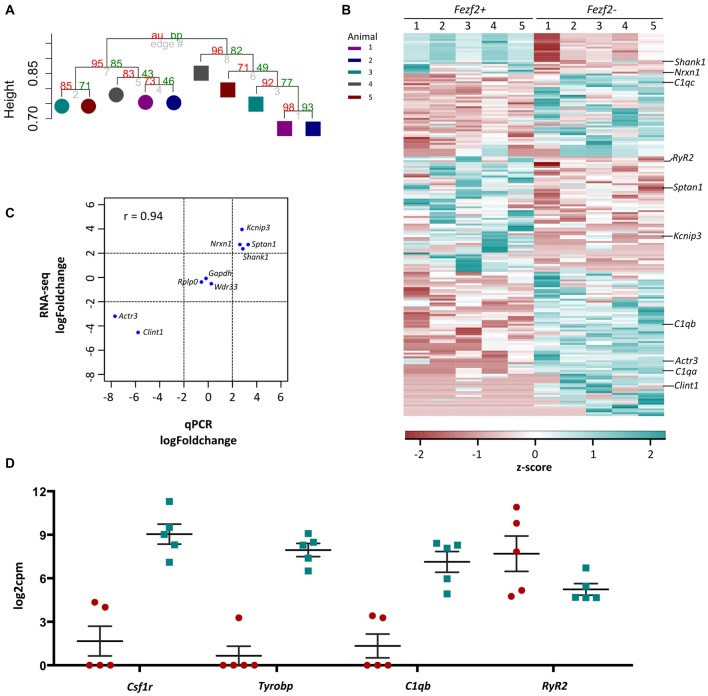
RNA-sequencing analysis of *Fezf2+ve* and *Fezf2−ve* IT-PNs. **(A)** Unsupervised correlation cluster analysis of the top 8% most variable genes across all samples. The approximately unbiased (au) *p*-value is shown as a percentage in red and the bootstrap (bp) *p*-value is in green. The *Fezf2+ve* and *Fezf2−ve* IT-PNs separate into two main clusters with an AU *p*-value > 95. Color key indicates the pairing of samples based on the animal from which they were isolated. **(B)** Heatmap of all significantly changed genes identified between *Fezf2+ve* and *Fezf2−ve* IT-PN samples. Relative changes in gene expression are represented by the z-score of transformed counts for each individual sample. **(C)** Pearson’s correlation analysis of the log fold change for nine genes as analyzed by qPCR data and RNA-seq. A significant positive relationship was observed (*p* = 0.0002). **(D)** Graph shows the log2cpm in *Fezf2+ve* and *Fezf2−ve* IT-PNs of genes of interest, including several microglia-associated genes.

Having assessed the quality of transcriptome data for *Fezf2+ve* and *Fezf2−ve* IT-PNs, we next wanted to determine, in an unbiased manner, whether there was any distinct clustering of samples. In order to do this, we performed unsupervised hierarchical clustering on the top 8% most variable genes across all 10 samples, irrespective of the IT-PN type. This analysis separated the IT-PN samples into two distinct clusters based on the presence or absence of *Fezf2* expression, with an AU *p*-value of >95 for both clusters (Figure 4A). The animal from which the samples were isolated did not influence the grouping. This result highlights the clear molecular differences between the IT-PNs.

### Differential Expression Analysis of *Fezf2+ve* and *Fezf2−ve* IT-PN RNA-Seq Data Identifies Significant Changes in Their Gene Expression Profiles

The clustering analysis indicated distinct differences in the transcriptome profiles of the *Fezf2+ve* and *Fezf2−ve* IT-PN types. To identify the genes that drove this separation, the RNA-sequencing data was analyzed for any significant changes in expression comparing the *Fezf2+ve* to the *Fezf2−ve* IT-PNs (*n* = 5 of each). Differential expression analysis of the 7,410 genes detected across all IT-PN samples revealed 199 genes that had significantly changed expression between the two IT-PN types (FDR ≤ 0.1, LFC ≥ 2). Of these genes, 118 had increased expression in the *Fezf2−ve* IT-PNs and 81 had increased expression in the *Fezf2+ve* IT-PNs (Figure 4B; Supplementary Table S4).

In order to technically validate the gene expression changes identified by RNA-seq, we performed qPCR on the pre-amplified cDNA from *Fezf2+ve* and *Fezf2−ve* IT-PNs. We analyzed nine genes (*n* = 3 or 4 mice for each IT-PN type), including three genes with unchanged expression (*Gapdh, Wdr33* and *Rplp0*), four genes that had significantly increased expression in *Fezf2+ve* IT-PNs (*Kcnip3, Sptan1, Shank1* and *Nrxn1*) and two genes that had significantly increased expression in *Fezf2−ve* IT-PNs (*Actr3* and *Clint1*). A significant correlation was observed between the log-fold changes in expression identified by qPCR when compared to the RNA-sequencing data (*r* = 0.93, *p* < 0.001; Figure 4C).

### Term Enrichment Analysis Reveals Distinct Functional Roles for the Unique Gene Expression Profiles of *Fezf2+ve* and *Fezf2−ve* IT-PNs

*Fezf2+ve* and *Fezf2−ve* IT-PNs have distinct morphological and electrophysiological differences (Tantirigama et al., 2014). Analysis of differentially expressed genes could reveal genes of interest that may be important for these unique functional differences. We used DAVID to analyze genes with either increased expression in *Fezf2+ve* or *Fezf2−ve* IT-PNs, separately.

**Figure 5:**
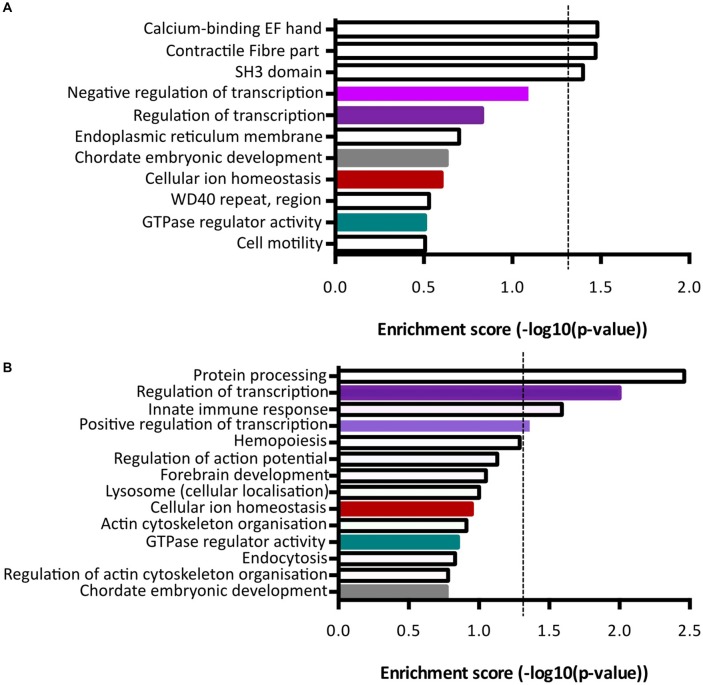
Term enrichment analyses of *Fezf2+ve* or *Fezf2−ve* IT-PN enriched genes. Genes that were enriched in *Fezf2+ve* and *Fezf2−ve* IT-PNs were analyzed separately for term enrichment **(A)** *Fezf2+ve* IT-PN enriched genes and **(B)** *Fezf2−ve* IT-PN enriched genes. The red dashed line indicates the significance cut off point (≥1.3), which is equivalent to *p*-value ≤ 0.05. Clusters common to both IT-PN types are color-coded accordingly. Shades of purple indicate clusters that all have a common role in transcription.

Functional annotation clustering of genes increased in *Fezf2+ve* IT-PNs identified three clusters with significant enrichment (−log10 *p*-value ≥ 1.3; Figure 5A; Supplementary Table S5). The top cluster was an enrichment of mRNAs encoding proteins that contain an EF-hand calcium-binding domain. Genes included in this category were *Kcnip3, Nrxn1, Sptan1, RyR2, Ppp3r1* and *S100a16*. The first three of these were included in the qPCR validation of differential expression (Figure 4C), with the increased expression in *Fezf2+ve* IT-PNs confirmed. Additionally, we observed enrichment in genes associated with contractile fiber (*Sptan1, Jup* and *RyR2*) and mRNAs encoding proteins containing an SH3 domain (*Bzrap1, Srgap1, Sptan1, Ostf1, Vat1l* and *Shank1*). All of these annotation clusters were unique to the *Fezf2+ve* IT-PN increased genes.

Analysis of genes with increased expression in *Fezf2−ve* IT-PNs revealed four clusters with significant enrichment (Figure 5B; Supplementary Table S6). These include three processes essential for cell function; protein processing (*C1qa, C1qb, C1qc, Srgn* and *Spcs2*), transcription regulation (*Atf4, Zfp36, Med12l, Zfhx3, Klf3, Egr1, Ikzf1, Btg2, Crebbp, Tnfrsf1b, Foxp2, Mitf, Selt, Zmiz1, Zf169, Arid3b, Runx1, Ncoa3, Tfdp2* and *Kdm2b*) and positive regulation of transcription (*Atf4, Klf3, Mitf, Efr1, Ikzf1, Zmiz1, Runx1* and *Crebbp*). Similarly, transcriptional regulation was an annotation cluster identified amongst the genes that were increased in *Fezf2+ve* IT-PNs, however this cluster was not significantly enriched (*Trps1, Zfhx4, Arhgap35, Mllt1, Kcnip3, Tada2a, Epas1, Tcf4, Nrg1, Pbx1, Per*3 and *Lrrfip*; Figure 5A). The presence of a transcriptional regulation cluster amongst both *Fezf2+ve* and *Fezf2−ve* enriched genes could indicate important regulatory genetic factors unique to each IT-PN type.

Another significantly enriched cluster of interest, unique to the genes with increased expression in *Fezf2−ve* IT-PNs, was a role in the biological process; innate immune response (*C1qa, C1qb, C1qc, Wdr43* and *Tnfrsf1b*). A number of genes that are considered enriched in microglia were amongst the genes with increased expression in *Fezf2−ve* IT-PNs, including *C1q*, *Csf1r, Tyrobp* (Figure 4D; Zhang et al., 2014) and *Siglech* (Kopatz et al., 2013). Whilst these IT-PNs display expression of classically microglial-associated genes, analysis of enriched GO terms amongst the top expressed genes in our samples were accordingly associated with neuronal processes (Supplementary Table S7). We further compared the GO terms identified from the top expressed genes of layer 5 neurons and microglial cells from Zeisel et al. (2015). This revealed a common overlap in neuronal associated enriched GO terms between our data and the layer 5 neurons. Importantly a role in immune system process was enriched amongst the top genes expressed in microglia, but did not overlap with either our dataset or the layer 5 neurons (Supplementary Table S7). Moreover, *C1q*, *Csf1r* and *Tyrobp* were recently identified as a cluster of genes with unique expression in a group of IT-PNs that project their axons transcallosally (Molyneaux et al., 2015). Together, this supports the unexpected finding of enriched immune-related gene expression in the *Fezf2−ve* IT-PNs.

### Immunohistochemistry of RYR2 Expression in Layer 5 IT-PNs Validates RNA-Seq Data

**Figure 6:**
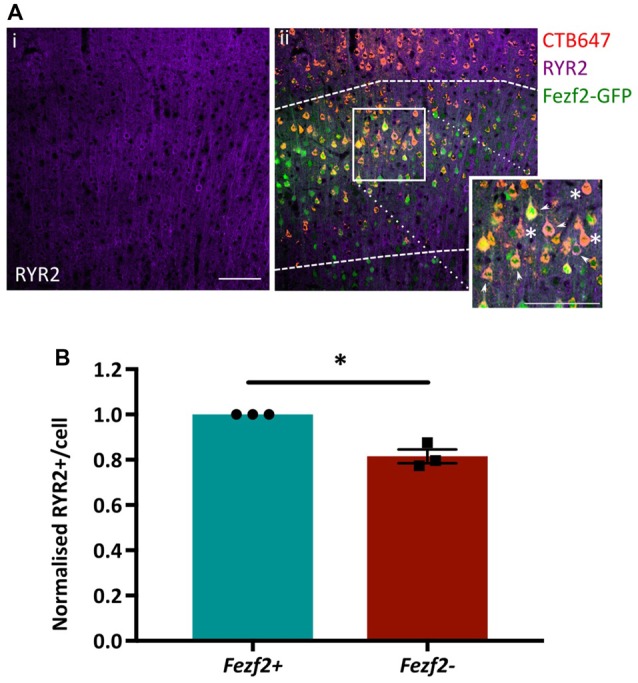
Validation of enriched RYR2 expression in *Fezf2+ve* IT-PNs by immunohistochemistry. Coronal sections from CTB647 injected *Fezf2-Gfp* mouse were labeled for Ryanodine receptor 2 (RYR2) expression and images captured in M1. **(Ai)** RYR2 expression in M1. **(ii)** Image shows co-expression of RYR2 (purple) with *Fezf2+ve* IT-PNs (green and red; arrowhead) and *Fezf2−ve* IT-PNs (red; asterisk). Dashed lines indicate layer 5 from which cells were selected for quantification. Scale bar is 100 μm. **(B)** Normalized percentage area of cell body labeled for RYR2 expression in *Fezf2+ve* and *Fezf2−ve* IT-PNs. There is a significant decrease of RYR2 protein expression in *Fezf2−ve* IT-PN cell bodies (*p* < 0.05, paired *t*-test, *n* = 3; ± SEM).

Functional annotation indicated an importance for calcium flux in *Fezf2+ve* IT-PNs (Figure 5A). Accordingly, the tufted appearance of *Fezf2+ve* IT-PNs (Tantirigama et al., 2014) suggests enhanced calcium flux associated with the dendritic calcium spikes seen in layer 5 cortical neurons (Schiller et al., 1997) and the genes identified in this cluster could therefore be particularly important to the phenotype of this IT-PN type. For example, RyR2 is involved in calcium induced calcium release from internal stores (Adasme et al., 2011) and could enhance calcium flux in *Fezf2+ve* IT-PNs. We were therefore interested in validating the differential expression of this particular mRNA (Figures 4B,C) at the protein level. RYR2 expression in M1 tissue was observed in the cytoplasm of the cell soma and within the dendrites (Figure 6Ai). Our purification of IT-PNs for RNA-seq isolated only the cell bodies, therefore we focused on quantifying the RYR2 expression within the cell bodies of *Fezf2+ve* (GFP and CTB647-positive) or *Fezf2−ve* (CTB647-positive) IT-PNs. Expression of RYR2 was detected in both *Fezf2+ve* and *Fezf2−ve* IT-PNs of layer 5 M1 (Figure 6Aii), however RYR2 expression was significantly reduced in *Fezf2−ve* IT-PNs, compared with *Fezf2+ve* IT-PNs (−18.5%; *p* < 0.05, paired *t*-test, *n* = 3; Figure 6B).

## Discussion

Our recent identification of two distinct IT-PN types in layer 5 M1 indicated common PN types that likely have unique roles in the M1 circuitry (Tantirigama et al., 2014). Here, we applied FACS and low-input RNA-seq methods to identify the transcriptome profiles that underpin these distinct IT-PN types. We demonstrate that there are clear differences in the transcriptome profiles of the *Fezf2−ve* and *Fezf2+ve* IT-PN types (Figure 5A) and identify 199 genes that have significantly changed expression. Importantly, we provide technical (qPCR) and separate biological validation of RYR2 protein expression, which provides support for the accuracy of this RNA-seq platform in identifying gene expression changes from low-RNA input. Additionally, we used term enrichment analysis of the enriched gene sets from *Fezf2+ve* and *Fezf2−ve* IT-PNs to investigate the functional implications. Together these analyses reveal the unique gene expression profile of these two IT-PN types and identify a number of interesting targets for future investigation. As only one protein target has been validated here, future validation of targets identified would strengthen the functional impact of these findings.

The analysis of transcript composition of RNA from the IT-PN samples demonstrated a high rate of mapping to intronic regions. As the mapping to intergenic regions was not greater than expected (Adiconis et al., 2013), this was unlikely to be genomic DNA contamination. The excessive intronic sequence could be a result of unspliced pre-mRNA present in the sample and certainly this high level of intronic mapping was observed in studies sequencing RNA isolated from neuronal nuclei (Lake et al., 2016). However, when a mixed population of cells were isolated from M1 tissue, mapping to intronic regions was significantly lower, suggesting the observation is unique to neuronal populations. Dueck et al. (2015) found that, particularly with cortical pyramidal neurons, there were increased rates of mapping to non-coding regions (>60%). Furthermore, intronic sequences are known to be important for transporting mRNA to the dendrites (Buckley et al., 2011), indicating a biological importance for retaining intronic sequences in neuronal mRNAs. Alternatively, the high mapping to introns in neurons could reflect a higher proportion of cytoplasmic poly-adenylated non-coding RNAs transcribed from intronic regions, which are considered to play an important maintenance role in the brain (Quan et al., 2017). Regardless of the source, our data, supported by findings in the literature, indicates that a high proportion of intronic regions are an accurate representation of neuronal RNA biology.

Functional analysis of M1 layer 5 IT-PN phenotypes revealed stark differences in the functional phenotypes of *Fezf2+ve* and *Fezf2−ve* IT-PNs (Tantirigama et al., 2014). A number of unique features in the *Fezf2+ve* IT-PN phenotype indicated a difference in calcium flux in these neurons. For example, active calcium signals are generated in the apical tuft (Bar Ilan et al., 2011), a morphological feature of *Fezf2+ve* IT-PNs, but absent in *Fezf2−ve* IT-PNs. Furthermore, *Fezf2+ve* IT-PNs also display a wider action potential (Tantirigama et al., 2014), indicative of enhanced calcium influx (Schiller et al., 1997; Kim et al., 2005; Bean, 2007). Here, our analysis of the *Fezf2+ve* gene expression profiles showed a significant enrichment of protein-encoding mRNAs that contain an EF-hand calcium-binding domain, which provides further support for a difference in the calcium flux of the *Fezf2+ve* IT-PNs. *RyR2* was a *Fezf2+ve* IT-PN enriched gene identified within this EF-hand domain annotation cluster. RYR2 is a calcium channel that mediates release of intracellular calcium stores from the endoplasmic reticulum via calcium-induced calcium release (Adasme et al., 2011), which could be critical for regenerative active calcium signaling in the tufts of *Fezf2+ve* IT-PNs (Schiller et al., 1997). Additionally, RYR2-mediated calcium release is important for synaptic plasticity (Adasme et al., 2011), with RYR2 and RYR3 required for bone derived neurotrophic factor-stimulated dendritic spine re-modeling (Adasme et al., 2011). RYR2 could be a key player in maintaining the unique apical tuft morphological feature, observed in *Fezf2+ve* IT-PNs. Here, we demonstrate biological validation of increased RYR2 protein expression in *Fezf2+ve* IT-PNs. In the future, it would be intriguing to investigate the effects of reducing RYR2 protein expression on the *Fezf2+ve* IT-PN functional phenotypes.

In the *Fezf2−ve* IT-PNs 118 genes had increased expression. Surprisingly, term enrichment analysis identified overrepresentation of genes associated with the innate immune response. Genes associated with this cluster included those encoding the three C1q chains (a–c). These form the C1q protein, which can bind directly to an antigen and trigger the classical complement cascade (Stevens et al., 2007). The C1q genes and several other genes enriched in *Fezf2−ve* IT-PNs (*Csf1r, Tryobp* and *Siglech*) are related to the immune system and are enriched in microglia (Kopatz et al., 2013; Zhang et al., 2014). However, despite a generalized association of these factors with microglia in the brain, expression of such genes also occurs in neurons (Stevens et al., 2007; Luo et al., 2013; Molyneaux et al., 2015; Guan et al., 2016), though comparatively at lower levels (Mancarci et al., 2017). A recent study performed extensive RNA-sequencing analysis on isolated callosal PNs, corticospinal PNs and cortico-thalamic PNs. Interestingly, the *C1q*, *Csf1r, Tyrobp* and *Siglech* genes were all identified in a cluster of genes with a similar pattern of enriched expression in CPNs (Molyneaux et al., 2015). Moreover, single-cell RNA-seq data from the S1 demonstrates that the same genes are detected almost uniquely in layer 5a pyramidal neurons, compared to other pyramidal neurons (Zeisel et al., 2015). Here, the isolation of *Fezf2−ve* IT-PNs from layer 5a of M1 has allowed the novel identification of enriched microglial-associated gene expression in a specific subset of IT-PN. The role for many of these immune-related genes in neurons has not yet been investigated. However, recent work suggests neuronal expression of C1q proteins is important in targeting synapses for microglial elimination (Stevens et al., 2007; Chu et al., 2010; Schafer et al., 2012; Bialas and Stevens, 2013; Hong et al., 2016). *Fezf2−ve* IT-PNs demonstrate a less complex morphology, lacking the apical tuft observed in *Fezf2+ve* IT-PNs (Tantirigama et al., 2014). It will be interesting to investigate whether C1q expression is important for the refined *Fezf2−ve* IT-PN morphology.

## Conclusion

We have successfully applied FACS-purification and low-input RNA-sequencing methods to identify the unique molecular profiles of two distinct IT-PN types from layer 5 of the mature mouse M1. Moreover, basic validation of the low-input RNA-seq platform was provided here with qPCR and IHC analyses. Functional annotation of the differentially expressed genes suggest a difference in calcium handling in *Fezf2+ve* IT-PNs and highlight a number of microglia-associated genes, enriched in *Fezf2−ve* IT-PNs. Overall the work has identified a number of interesting candidates that may be important for generating and/or maintaining the unique phenotypes of these IT-PNs. This provides interesting target proteins for future validation and analysis, which will be essential for developing our understanding of neuronal maintenance in the mature M1 circuitry.

## Data Availability

GEO accession number for IT-PN RNA-sequencing data is GSE107586.

## Author Contributions

AC, RD, RE and SH: study concept and design. AC and RD performed the experiments and acquired data. AC, RE and SH: analysis and interpretation of the data. AC: drafted the manuscript. AC, SH, RE and RD: major critical revisions of the manuscript. All authors had full access to the manuscript and data for approval.

## Conflict of Interest Statement

The authors declare that the research was conducted in the absence of any commercial or financial relationships that could be construed as a potential conflict of interest.

## Footnotes

^1^https://github.com/najoshi/sabre
