# Supplementary material for: Transcriptome Profiling of Layer 5 Intratelencephalic Projection Neurons From the Mature Mouse Motor Cortex
Source: Front Mol Neurosci. 2018 Nov 12;11:410. doi: 10.3389/fnmol.2018.00410 (PMC6240696; doi:10.3389/fnmol.2018.00410)
Supplement: Supplementary file 1 [file Data_Sheet_1.DOCX]

# Transcriptome profiling of layer 5 intratelencephalic projection neurons from the mature mouse motor cortex

Alison J. Clare^1,3,4^, Robert C. Day^1,4^, Ruth M. Empson^2,3^ and Stephanie M. Hughes^1,3,4^*.

Departments of ^1^Biochemistry and ^2^Physiology, ^3^Brain Health Research Centre, ^4^Genetics Otago, School of Biomedical Sciences, University of Otago, Dunedin, New Zealand 9054.

* **Correspondence**:

Stephanie M. Hughes

[stephanie.hughes@otago.ac.nz](mailto:stephanie.hughes@otago.ac.nz)

## Supplementary material

**
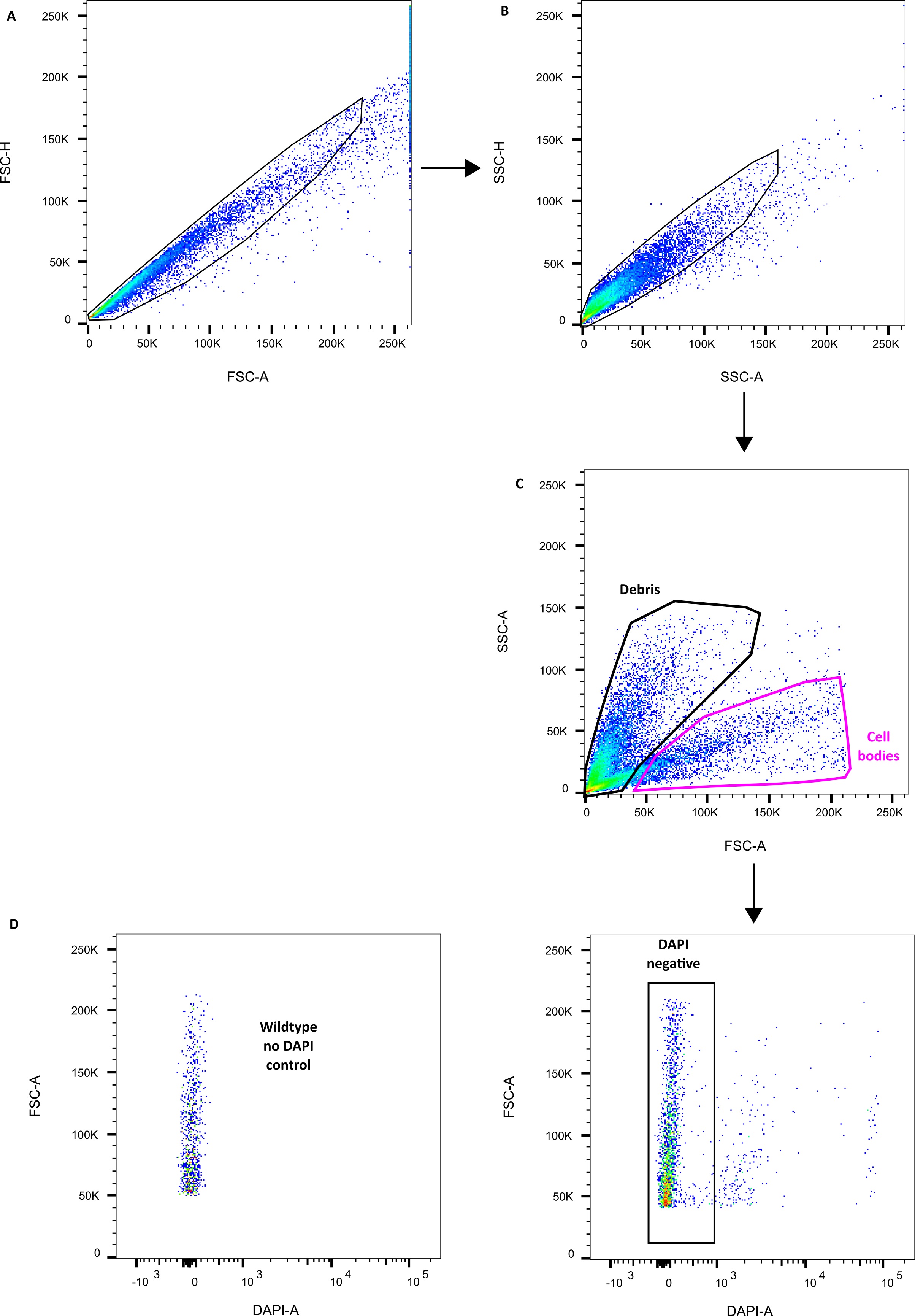
**

**Supplementary Figure 1 Example of gating applications to determine live viable single cell population for isolation of the cells of interest.** Cells of the same size should have similar area and height and thus plotting the light scatter of area and height will cluster as a diagonal correlation. Any events (dots) that cluster away from this line will be doublets or cell clumps. Gates were set according to these using the forward scatter (FSC) (A) and side-scatter (SSC) **(B)**. **(C)** The FSC and SSC were plotted together to separate cell bodies from debris. Debris is generally smaller in size (FSC) with greater granularity (SSC; Guez-barber et al. 2011). **(D)** The final gating stage was set to determine the live cell population. A gate was set to exclude the DAPI-positive cells (dead cells; DAPI-negative), according to the wildtype (DAPI-negative) sample. Final sorting of samples was taken from this single cell, live viable population.

**
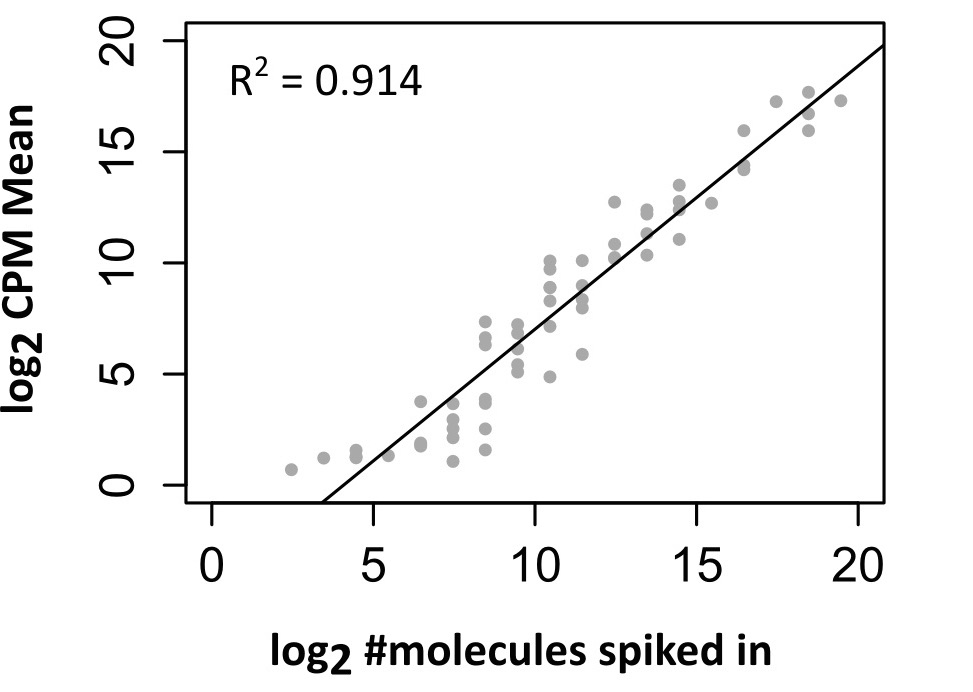
**

**Supplementary Figure 2 Linear regression analysis of ERCC spike-in.** Linear regression analysis of the ERCC reads compared to the known molecules spiked in (R^2^ = 0.914; p < 2.2e-16).


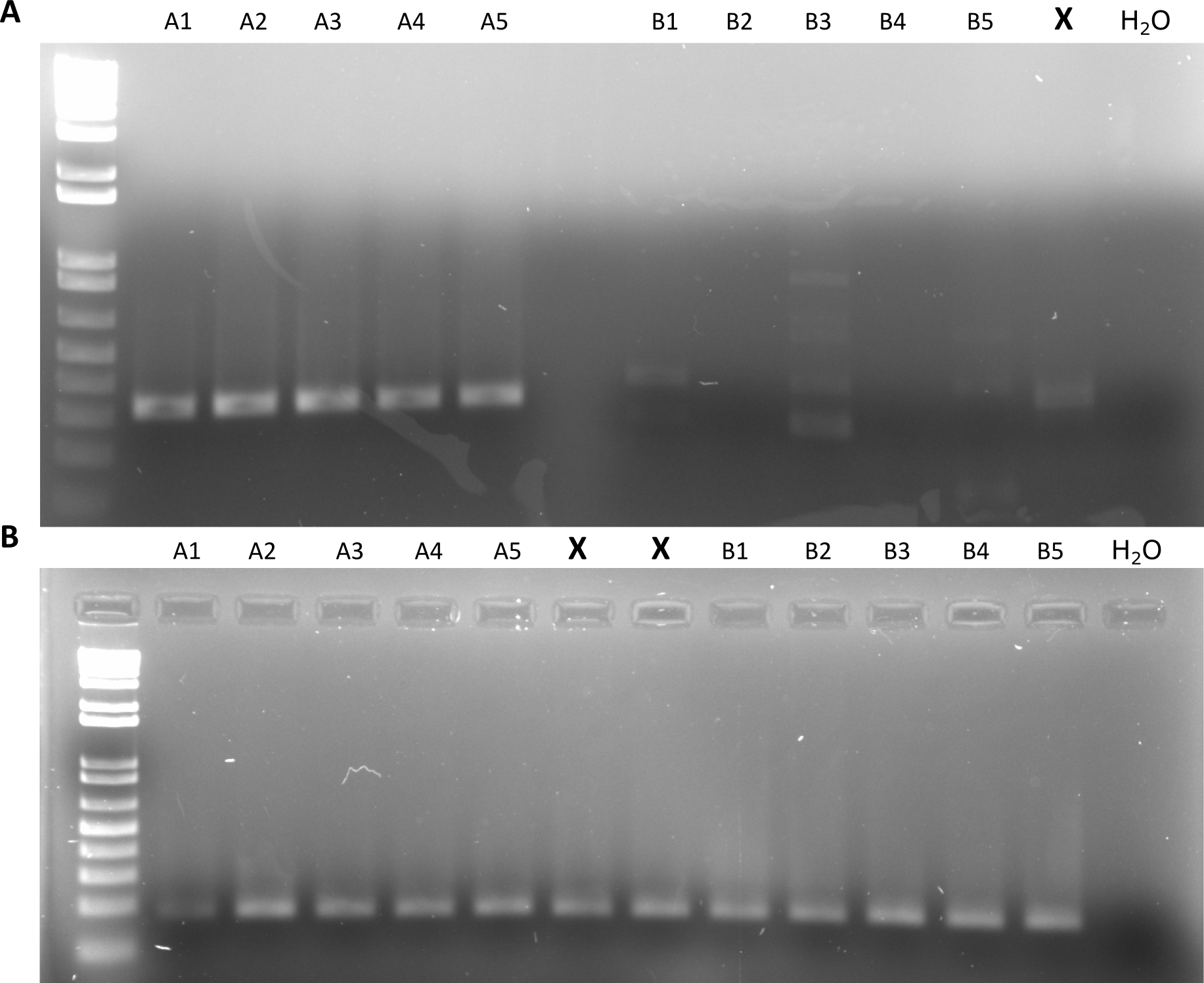


**Supplementary Figure 3** Original agarose gels displaying PCR products of endogenous *Fezf2* **(A)** and *Gapdh* **(B)**. A1 – A5 are *Fezf2+ve* IT-PN samples from animals 1 – 5. B1 – B5 are *Fezf2-ve* samples from animals 1 – 5. An X indicates a sample run that is not related to the IT-PN samples. H_2_O indicted the negative template control. Samples were run on 1.5% agarose gel at 100 volts for 35 min with a 1kb plus ladder (Invitrogen, NZ).

**Supplementary table 3**. Mean (± standard error of the mean) of mapping rates to exonic, intronic and intergenic regions and results of two-way ANOVA with Tukey’s multiple comparisons test.

|  | *Fezf2+ve* | *Fezf2-ve* | Mixed | p-value | | |
| --- | --- | --- | --- | --- | --- | --- |
|  |  |  |  | ***Fezf2+ve* vs. *Fezf2-ve*** | ***Fezf2+ve* vs. Mixed** | ***Fezf2+ve* vs. Mixed** |
| Exonic | 38.5 (± 7.6) | 31.9 (± 4.1) | 68.6 (± 4.0) | ns | 0.0012 | 0.0001 |
| Intronic | 51.5 (± 7.0) | 56.9 (± 4.7) | 18.4 (± 3.5) | ns | 0.0004 | <0.0001 |
| Intergenic | 9.9 (± 1.0) | 11.2 (± 2.3) | 13.0 (± 1.6) | ns | ns | ns |
